# Phospholipid scrambling by a TMEM16 homolog of *Arabidopsis thaliana*

**DOI:** 10.1111/febs.16279

**Published:** 2021-11-26

**Authors:** Anna Boccaccio, Cristiana Picco, Eleonora Di Zanni, Joachim Scholz‐Starke

**Affiliations:** ^1^ Institute of Biophysics Consiglio Nazionale delle Ricerche Genova Italy; ^2^ Present address: Department of Anesthesiology Weill Cornell Medical College New York NY USA

**Keywords:** anoctamin, endoplasmic reticulum, phospholipid, scramblase, TMEM16

## Abstract

Membrane asymmetry is important for cellular physiology and established by energy‐dependent unidirectional lipid translocases, which have diverse physiological functions in plants. By contrast, the role of phospholipid scrambling (PLS), the passive bidirectional lipid transfer leading to the break‐down of membrane asymmetry, is currently still unexplored. The *Arabidopsis thaliana* genome contains a single gene (At1g73020) with homology to the eukaryotic TMEM16 family of Ca^2+^‐activated phospholipid scramblases. Here, we investigated the protein function of this Arabidopsis homolog. Fluorescent AtTMEM16 fusions localized to the ER both in transiently expressing Arabidopsis protoplasts and HEK293 cells. A putative scrambling domain (SCRD) was identified on the basis of sequence conservation and conferred PLS to transfected HEK293 cells, when grafted into the backbone of the non‐scrambling plasma membrane‐localized TMEM16A chloride channel. Finally, AtTMEM16 ‘gain‐of‐function’ variants gave rise to cellular phenotypes typical of aberrant scramblase activity, which were reversed by the additional introduction of a ‘loss‐of‐function’ mutation into the SCRD. In conclusion, our data suggest AtTMEM16 works as an ER‐resident lipid scramblase in Arabidopsis.

## Graphical abstract

Scramblases allow the passive bidirectional transmembrane lipid transfer leading to the break‐down of membrane asymmetry. Here, we investigated the protein localization and function of AtTMEM16, the *Arabidopsis thaliana* homolog of the eukaryotic TMEM16 family of Ca^2+^‐activated phospholipid scramblases. We show that AtTMEM16 works as an ER‐resident lipid scramblase.

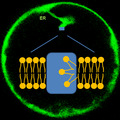

**Abbreviations**
ALA: aminophospholipid ATPase
ER: endoplasmic reticulum
PLS: phospholipid scrambling
PM: plasma membrane
PS: phosphatidylserine
SCRD: scrambling domain

## Introduction

Providing a physical boundary towards the external environment and between the different compartments of eukaryotic cells, biological membranes are important interfaces for the exchange of matter, energy and information. As such, their lipid and protein composition is tailored to their specific physiological functions and determines their identity. Membrane lipids show significant chemical and organizational complexity. Individual lipid species of a membrane vary in their amount, in their localization in subdomains within a membrane leaflet, for example, lipid rafts or nanodomains, and finally also in their relative distribution between the two leaflets of the bilayer. This membrane asymmetry is an important prerequisite for many cellular processes, for example, for the generation of membrane curvature during vesicle‐mediated membrane trafficking, membrane‐based signalling events and the recruitment of proteins to specific membrane domains or organelles. In plants, anionic phospholipids like phosphatidylinositol‐4‐phosphate (PI4P) and phosphatidylserine (PS) are highly enriched at the cytosolic face of the plasma membrane and, to a lower extent, down the endocytic pathway [1, 2]. It has been proposed that the graded distribution of negative charge provided by their anionic head groups determines an electrostatic territory attracting positively charged interactors from the cytosol [3, 4, 5].

The establishment and maintenance of phospholipid asymmetry is an energy‐consuming process. The spontaneous inter‐leaflet movement of phospholipids is very slow [6], since the translocation of the polar head groups across the hydrophobic membrane core is energetically unfavourable. *Lipid flippases* are primary active transporters moving phospholipids against a concentration gradient to the cytosolic side of the membrane. Most flippases belong to the P4 ATPase subfamily of P‐type ATPases and plant genomes generally contain a large number of P4 ATPase homologs [7], and also in the model plant *Arabidopsis thaliana* 12 family members have been identified [8], which were named aminophospholipid ATPases (ALA). Based on the analysis of gene knock‐out mutant plants, ALA proteins have been implicated in developmental processes, cellular signalling, defence responses, and adaptation to temperature changes (recently reviewed by Nintemann *et al*., [9]). As noted by these authors, such pleiotropy in flippase mutant phenotypes might be indicative of a non‐specific impairment of cellular lipid homeostasis and/ or vesicle trafficking. The widespread expression pattern of ALA proteins and their localization in the plasma membrane as well as different endomembrane systems [9], consistent with an asymmetric character for these membranes, support the view that membrane asymmetry may be the default state in plant cells.

Just as the asymmetric distribution of phospholipids has functional relevance for specific cellular processes, so has also the rapid regulated break‐down of lipid asymmetry (lipid scrambling). For comparison, the transmembrane proteins mediating this passive equilibrative lipid transport (*lipid scramblases*) in this context correspond to passive ion channels harvesting the energy contained in the electrochemical gradient created by primary active ion pumps (which in turn are analogous here to lipid flippases). In animal cells, the extracellular exposure of PS, normally confined to the internal leaflet of the plasma membrane, is a critical signal involved in blood coagulation or the recognition of apoptotic cells [10]. At present, the available evidence for lipid scrambling in plants is very limited. In suspension‐cultured tobacco cells, induction of apoptosis evoked the binding of the PS‐marker annexin‐V at the cell surface [11], which suggests extracellular PS exposure may occur also in plants. Furthermore, constitutive and passive phospholipid translocation activity was identified in spinach ER membrane extracts [12], which may serve to maintain membrane symmetry and stability, since the *de novo* synthesis of phospholipids occurs at the cytoplasmic leaflet. No specific gene product has been assigned to these activities.

TMEM16 proteins (also named anoctamins) are a family of integral membrane proteins present in all eukaryotes. Several members of animal and fungal origin have been shown to function as Ca^2+^‐dependent lipid scramblases [13, 14, 15, 16, 17, 18]. Most eukaryotic genomes contain only one or two TMEM16 homologs, while the mammalian subfamily has diversified into 10 members, some of which localize to the plasma membrane [17, 19] and others intracellularly [14, 15, 20]. Intriguingly, two of them, TMEM16A and TMEM16B, have lost their ability to scramble lipids and evolved into Ca^2+^‐dependent chloride channels, which are critically involved in different aspects of epithelial transport, smooth muscle function and neuronal signalling [21]. Since the chloride‐channel function has not been observed outside the mammalian subfamily, it may be legitimate to consider lipid scrambling the original function of TMEM16 proteins. During evolution, this transition from *lipid channel* to *ion channel* may have been facilitated by the fact that all recognized scramblases give rise to Ca^2+^‐activated non‐selective ion currents [15, 16, 22, 23, 24], most likely due to unspecific ion slippage along the lipid transport pathway. Structural analyses have shown that TMEM16 scramblases are homodimers with a double‐barrelled architecture, each monomer having 10 transmembrane‐spanning helical domains and an open groove exposed to the hydrophobic membrane core as a lipid conduction pathway [13, 14, 25].

The genome of the model plant *Arabidopsis thaliana* contains a single TMEM16‐like gene (locus At1g73020). Among other putative anion transporter candidates, AtTMEM16 has been included in a recent study investigating anion transport across the plasma membrane of Arabidopsis pollen tubes. Pollen tube protoplasts from *tmem16* knock‐out plants showed specific alterations of their membrane currents, which lead the authors to propose the AtTMEM16 protein as an anion/H^+^ cotransporter [26]. However, direct data on protein function in heterologous expression systems and on its subcellular localization are still missing. Here, we show that AtTMEM16 localizes to the ER membrane and likely functions as a Ca^2+^‐dependent lipid scramblase.

## Results and discussion

### The AtTMEM16 protein sequence presents features of Ca^2+^‐activated lipid scramblases

To obtain cues on the putative function of AtTMEM16, we first examined its predicted amino acid sequence. The *AtTMEM16* gene (At1g73020) contains an open reading frame encoding 665 amino acids. Overall sequence identity with TMEM16 proteins from other kingdoms is low (Fig. S1), showing 19.8% identity with Ist2p (946 aa) from *Saccharomyces cerevisiae*, 21.9% with afTMEM16 (735 aa) from *Aspergillus fumigatus*, 27.3% with human TMEM16A (986 aa), 27.4% with human TMEM16E (913 aa) and 25.5% with human TMEM16K (660 aa). Important identifying features of mammalian TMEM16 proteins are their cytosolic calcium dependence and their functional dichotomy into chloride channel and phospholipid scramblase branches. The ability to scramble in the latter has been nailed down to a short protein stretch spanning the cytosolic face membrane between TM4 and TM5, denominated scrambling domain (SCRD) [18]. Notably, using SCRD swapping it has been possible to convert the non‐scrambling TMEM16A chloride channel into a scrambler [18, 27].

**Fig. 1:**
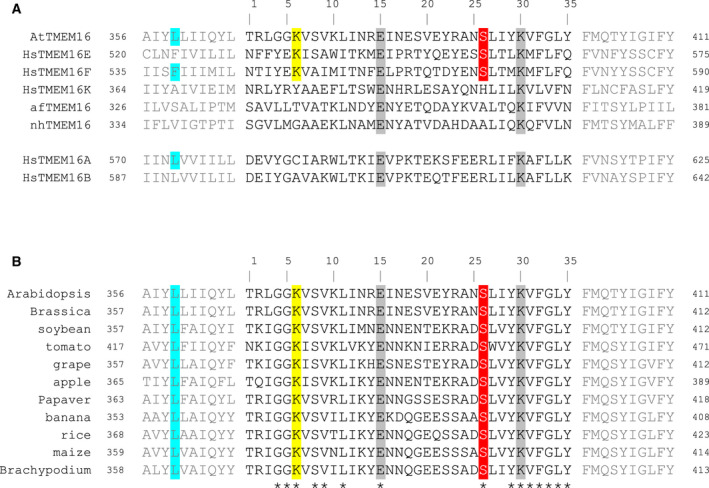
Scrambling domain sequence alignment of TMEM16 family members. Protein sequence alignments of the 35‐aa scrambling domain (SCRD) and adjacent regions (in grey letters) of AtTMEM16 with TMEM16 homologs of known function (A) and with TMEM16 homologs from different dicot and monocot plant species (B). In (A), sequences of the human lipid scramblases TMEM16E, TMEM16F and TMEM16K, the fungal lipid scramblases afTMEM16 and nhTMEM16, and the human Cl^‐^ channels TMEM16A and TMEM16B. In (B), sequences from *Brassica rapa* (*Brassicaceae*), *Glycine max* (soybean; *Fabaceae*), *Solanum lycopersicum* (tomato; *Solanaceae*), *Vitis vinifera* (grape; *Vitaceae*), *Malus domestica* (apple; *Rosaceae*), *Papaver somniferum* (*Papaveraceae*), *Musa acuminata* (banana; *Musaceae*), *Oryza sativa* (rice), *Zea mays* (maize) and *Brachypodium distachyon* (all three from *Poaceae*). Amino acid residues fully conserved among TMEM16 homologs are marked in grey. Two critical amino acid residues for HsTMEM16E scramblase function [28] are marked in yellow (lysine at position 6) and red (serine at position 26). Inner gate residues homologous to Phe518 in murine TMEM16F [36] are marked in cyan. Fully conserved SCRD amino acid residues within the plant TMEM16 group are indicated by an asterisk.

Sequence alignments of the SCRD homology region for several mammalian and fungal TMEM16 proteins of demonstrated function (Fig. 1A) showed that AtTMEM16 shares two amino acid residues, glutamate at SCRD position 15 and lysine at position 30, which are fully conserved in both scramblases and chloride channels. Gyobu *et al*. (2017) identified two key residues contributing to the scrambling activity of TMEM16E, also present in TMEM16F, that is, lysine at SCRD position 6 and serine at position 26. Both residues are well conserved among scramblases of the murine and human TMEM16 families, and TMEM16E mutants carrying the corresponding residues present in the chloride channel TMEM16A lost their scrambling activity [28]. Moreover, isoleucine substitution of the serine residue in human TMEM16E (Ser555Ile), related to limb‐girdle muscular dystrophy [29], a recessively inherited genetic disease, abolished both non‐selective ion transport and lipid scrambling [30]. Notably, both scrambling‐related residues are also present at the homologous positions of AtTMEM16 (Fig. 1A) and fully conserved in the SCRD sequences from ten further dicot and monocot plant species (Fig. 1B), suggesting that the respective amino acid positions were not subject to variation during recent plant evolution.

**Fig. 2:**
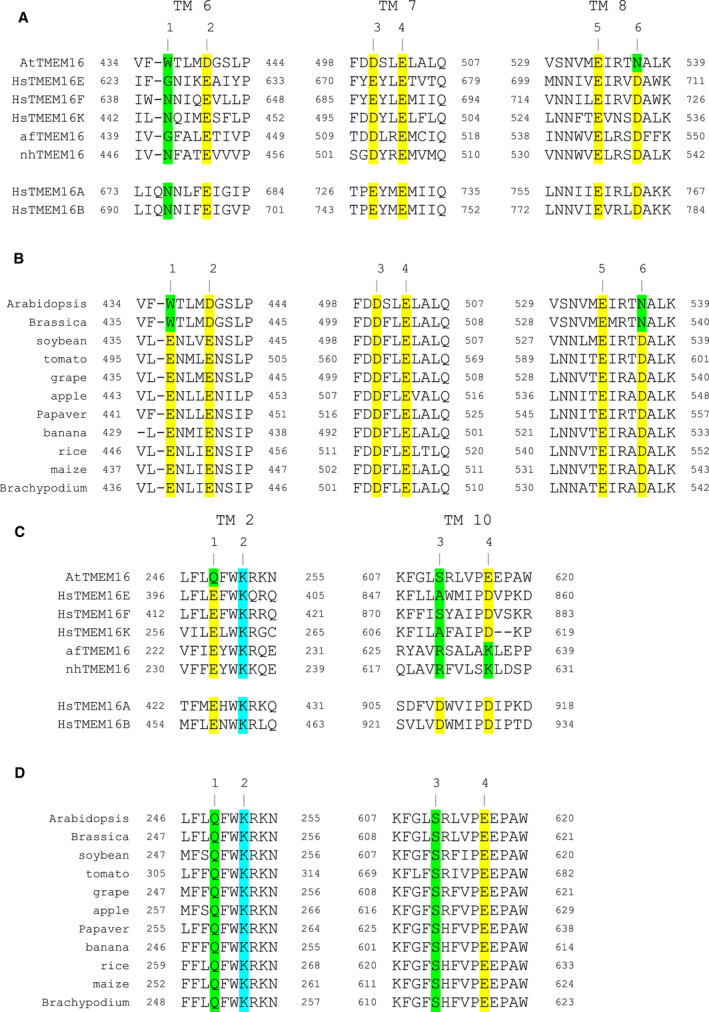
Sequence alignment for Ca^2+^ binding sites in TMEM16 proteins. (A, B) Primary cytosolic Ca^2+^‐binding site. Protein sequence alignments of the TM 6, 7 and 8 regions of AtTMEM16 with TMEM16 homologs of known function (A) and with TMEM16 homologs from different dicot and monocot plant species (B). Critical residues involved in Ca^2+^ coordination are indicated by positions 1 through 6. Conserved acidic residues are marked in yellow. Variable residues or deviations from conservation are marked in green. (C, D) Mammalian ‘third Ca^2+^‐binding site’. Protein sequence alignments of the TM 2 and 10 regions of AtTMEM16 with TMEM16 homologs of known function (C) and with TMEM16 homologs from different dicot and monocot plant species (D). Critical residues involved in Ca^2+^ coordination are indicated by positions 1 through 4. Conserved acidic residues at positions 1 and 4 are marked in yellow, the basic lysine residue at position 2 in cyan. Variable residues or deviations from conservation are marked in green. In (A) and (C), sequences of the human lipid scramblases TMEM16E, TMEM16F and TMEM16K, the fungal lipid scramblases afTMEM16 and nhTMEM16, and the human Cl^‐^ channels TMEM16A and TMEM16B. In (B) and (D), sequences from *Brassica rapa* (*Brassicaceae*), *Glycine max* (soybean; *Fabaceae*), *Solanum lycopersicum* (tomato; *Solanaceae*), *Vitis vinifera* (grape; *Vitaceae*), *Malus domestica* (apple; *Rosaceae*), *Papaver somniferum* (*Papaveraceae*), *Musa acuminata* (banana; *Musaceae*), *Oryza sativa* (rice), *Zea mays* (maize) and *Brachypodium distachyon* (all from *Poaceae*).

All TMEM16 proteins investigated so far require increased cytosolic Ca^2+^ concentrations for activation. The primary Ca^2+^‐binding pocket present in both fungal and animal homologs is formed by three pairs of amino acid residues located at the inner parts of TM domains 6, 7, and 8 [13, 14, 25, 31]. Sequence alignments of these regions showed that the four conserved acidic residues at positions 2‐5 (either glutamate or aspartate) were present also in AtTMEM16 (Fig. 2A). Conversely, AtTMEM16 carries a tryptophan residue at position 1, which generally displays some intra‐ and inter‐specific variability, and an asparagine residue instead of the highly conserved aspartate at position 6 (Fig. 2A). Interestingly, both these sequence variations in AtTMEM16 were confirmed in further species of the *Brassicaceae* family (with *Brassica rapa* shown in Fig. 2 being one example), but not in more distant plant relatives, which without exception carry a glutamate residue at position 1 and an aspartate residue at position 6 (Fig. 2B). In addition to this primary binding pocket for 2 Ca^2+^ ions, very recently a ‘third Ca^2+^ site’ has been identified in mammalian TMEM16A and TMEM16K [14, 32]. Also, this site appears to be well conserved in AtTMEM16 (Fig. 2C), albeit with a plant‐specific glutamate to glutamine exchange at position 1 (Fig. 2C‐D). In summary, the protein sequence of AtTMEM16 (and of plant TMEM16 homologs in general) shows key features of Ca^2+^‐activated lipid scramblases.

### AtTMEM16 shows ER localization in Arabidopsis mesophyll cells and HEK293 cells

**Fig. 3:**
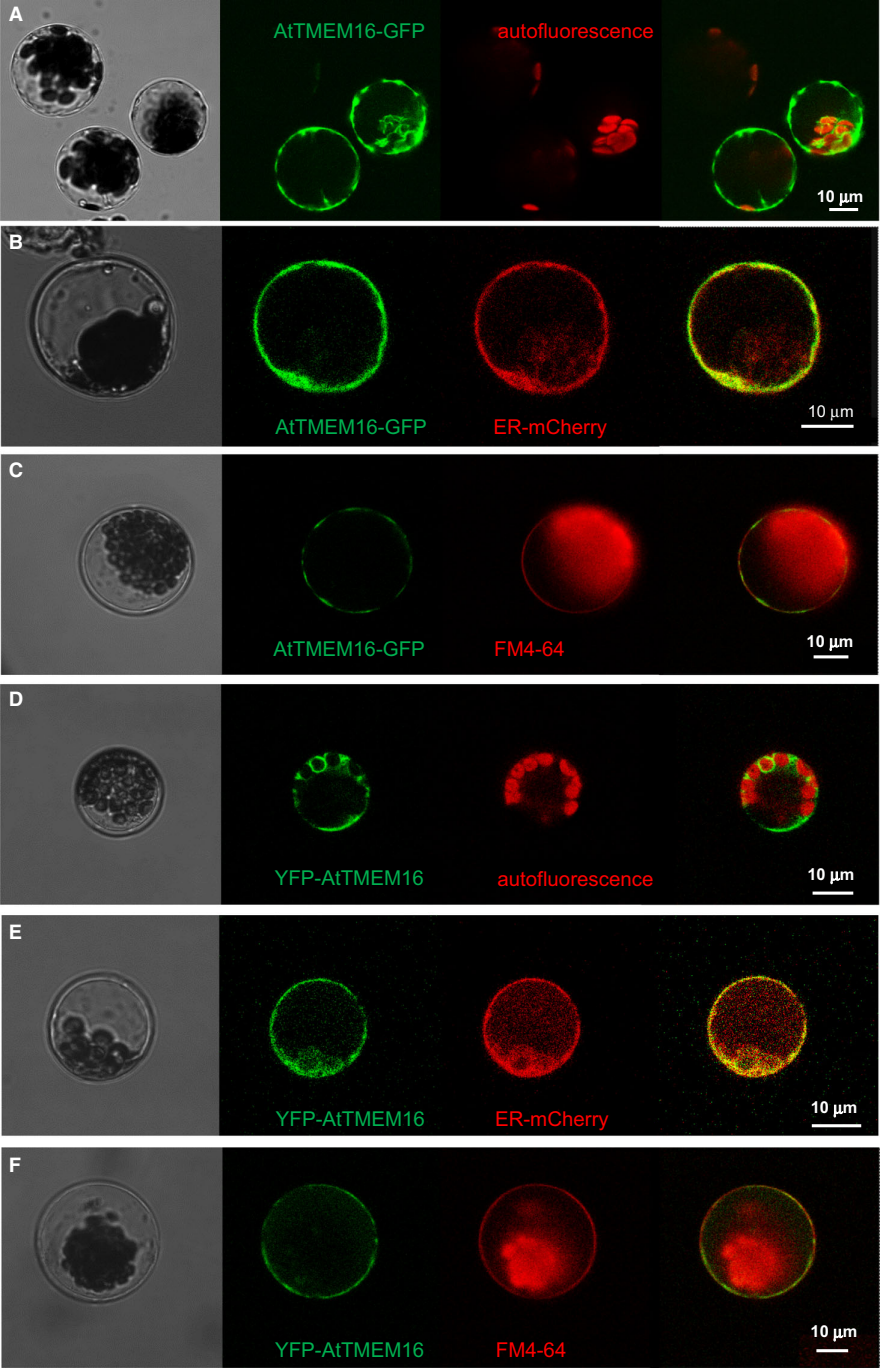
Endoplasmic reticulum localization of the AtTMEM16 protein in plant cells. Confocal images of Arabidopsis mesophyll protoplasts transiently expressing AtTMEM16‐EGFP (A–C) or YFP‐AtTMEM16 (D–F) fusion constructs. Chlorophyll autofluorescence was used to label chloroplasts (A, D), ER membranes were labelled by the mCherry‐HDEL marker (B, E) and plasma membrane staining was done by incubation with FM4‐64 dye (C, F). From left to right: transmission light, green channel (EGFP or YFP), red channel (autofluorescence, FM4‐64 or ER‐mCherry), merge of green and red channels.

Several members of the mammalian TMEM16 protein family are targeted to the plasma membrane (PM) [17, 19, 33], while others retain an intracellular localization [14, 15, 20]. To determine the subcellular localization of the plant homolog, fluorescently tagged AtTMEM16 proteins were transiently expressed in isolated Arabidopsis mesophyll cells. Confocal imaging showed that AtTMEM16‐EGFP expression gave rise to patchy fluorescence signals, which appeared mostly intracellular and were particularly intense in the regions surrounding the chloroplasts (Fig. 3A). The space occupied by the large lytic vacuole remained unlabelled in these protoplasts. Coexpression of the mCherry‐HDEL marker showed that this EGFP‐positive compartment corresponded largely to the endoplasmic reticulum (Fig. 3B). By contrast, no fluorescence overlap was observed in protoplasts stained with the PM marker FM4‐64 (Fig. 3C). Similar results were obtained with an AtTMEM16 fusion protein carrying yellow fluorescent protein (YFP) at its N‐terminus (Fig. 3D–F). For quantitative analyses of AtTMEM16 and ER marker co‐localization, we determined Manders' overlap coefficient representing the fraction of AtTMEM16‐containing pixels which were additionally positive for ER marker: M values were 0.69 ± 0.03 for AtTMEM16‐EGFP (*n* = 19) and 0.78 ± 0.04 for YFP‐AtTMEM16 (*n* = 22).

**Fig. 4:**
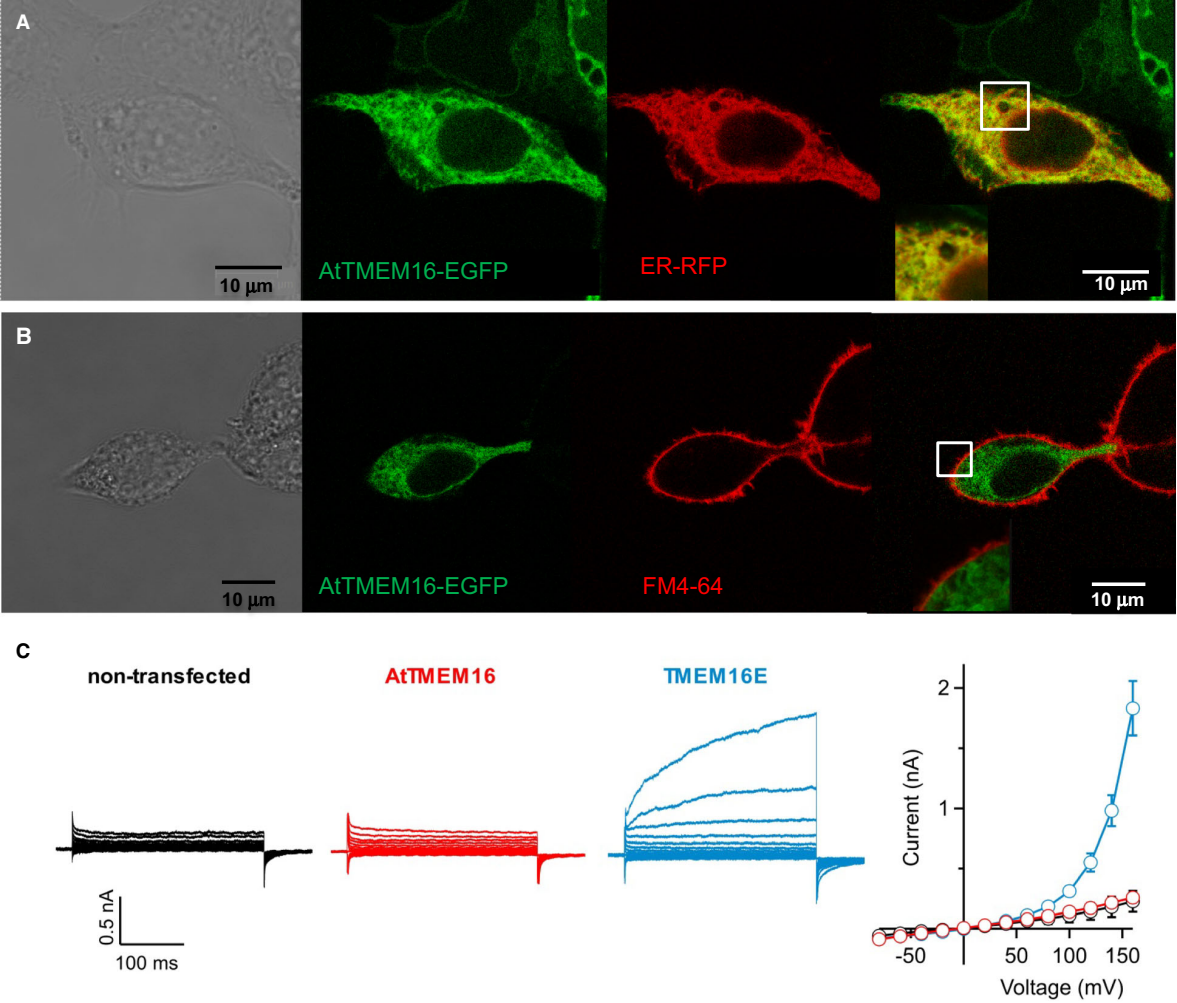
AtTMEM16 localizes to the endoplasmic reticulum in HEK293 cells. (A, B) Confocal images of HEK293 cells transiently transfected with the AtTMEM16‐EGFP fusion construct and co‐stained with the ER marker CellLight ER‐RFP (A) or with the PM marker FM4‐64 (B). From left to right: transmission light, green channel (EGFP), red channel (ER‐RFP or FM4‐64), merge of green and red channels. Insets: closeup views of the squared regions. (C) Whole‐cell patch‐clamp recordings on HEK293 cells transiently transfected with AtTMEM16‐EGFP (red traces) or HsTMEM16E‐EGFP (blue traces) constructs or non‐transfected control cells (black traces). The intracellular solution contained 3 µm calculated free Ca^2+^. The stimulation protocol consisted of 300‐ms voltage steps ranging from −80 to +160 mV with 20‐mV increments, followed by a 175‐ms tail pulse to −80 mV. Holding potential at 0 mV. Right panel: I‐V relationships (mean ± sem) for *n* = 8 non‐transfected cells, *n* = 9 AtTMEM16‐EGFP and *n* = 7 HsTMEM16E‐EGFP.

A number of studies have shown that human embryonic kidney (HEK293) cells represent a valid heterologous expression system for functional studies on TMEM16 ion channels and lipid scramblases [15, 24, 30, 34, 35, 36, 37, 38]. In transiently transfected HEK293 cells, EGFP‐tagged AtTMEM16 localized to intracellular membranes, showing near‐complete co‐localization with the ER marker CellLight ER‐RFP (Fig. 4A; M = 0.82 ± 0.03, *n* = 11). By contrast, no overlap with the FM4‐64 marker was apparent suggesting that AtTMEM16 is not trafficked to the PM in these cells (Fig. 4B). To test the possibility that a minor fraction of AtTMEM16 protein may still be present at the PM, we performed whole‐cell patch‐clamp recordings with an intracellular solution containing 3 µm free Ca^2+^, sufficient to activate non‐selective ion currents mediated by human TMEM16E (Fig. 4C), an intracellular lipid scramblase with partial PM localization in HEK293 cells [15]. However, in AtTMEM16‐EGFP expressing cells, current amplitudes were low and not significantly different from those seen in non‐transfected control cells (Fig. 4C), indicating that AtTMEM16 is not trafficked to the PM, as anticipated from confocal data, or alternatively, is unable to mediate ion currents under these conditions. Taken together, these data indicate that AtTMEM16 retains an intracellular localization in the ER compartment of both Arabidopsis mesophyll protoplasts and cultured HEK293 cells.

### The AtTMEM16 SCRD confers scrambling activity to the TMEM16A chloride channel

**Fig. 5:**
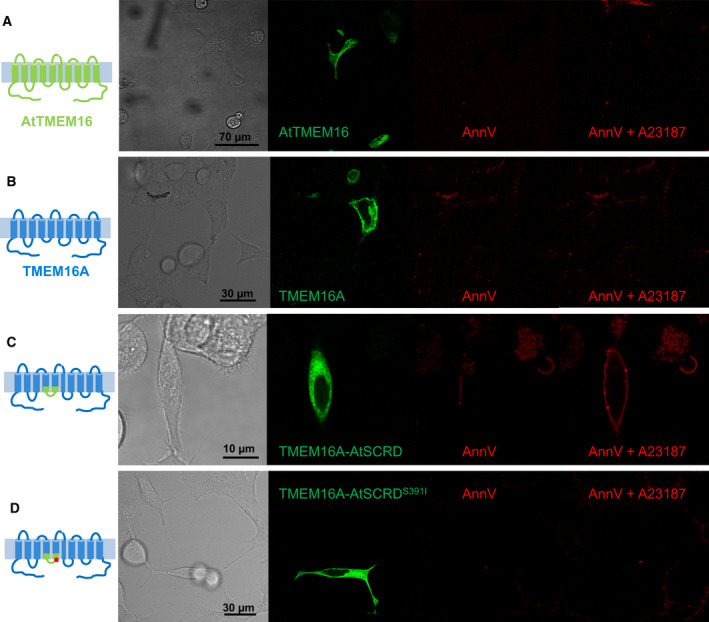
Lipid scrambling activity of a TMEM16A‐AtTMEM16 chimera. (A–D) Confocal images of HEK293 cells transiently transfected with AtTMEM16‐EGFP (A), TMEM16A‐EGFP (B), TMEM16A‐AtSCRD‐EGFP (C) or TMEM16A‐AtSCRD^S391I^‐EGFP (D) constructs and stained with Alexa555‐conjugated annexin‐V in the absence or presence of the Ca^2+^ ionophore A23187 (5 μm), as indicated. Representative images, similar results were obtained in two independent transfections. From left to right: cartoon depicting AtTMEM16 (in green), mouse TMEM16A (in blue) or TMEM16A‐AtSCRD chimera (mixed); transmission light image, green channel (EGFP), red channel (Alexa555).

Lack of PM localization precluded a direct investigation of the putative lipid scrambling function of AtTMEM16. We, therefore, applied an established chimeric approach [18, 27] consisting in swapping the 35‐aa SCRD homology region of AtTMEM16 (amino acids Thr366 to Tyr400; Fig. 1) into the backbone of the PM‐localized Ca^2+^‐activated chloride channel TMEM16A (denominated TMEM16A‐AtSCRD). We used HEK293 live‐cell assays of PM‐localized lipid scrambling activity, based on the binding of Alexa555‐conjugated annexin‐V to extracellularly exposed phosphatidylserine [15, 30]. To stimulate cytosolic Ca^2+^ increases required for TMEM16 protein activation, cells were acutely exposed to the Ca^2+^ ionophore A23187. No Alexa555‐related fluorescence signals were detected at the surface of AtTMEM16‐EGFP expressing cells in any condition (Fig. 5A), as expected from the above results showing AtTMEM16 absent from the PM (Fig. 4). Murine TMEM16A‐EGFP fusion protein localized to the PM, as reported earlier [19], but was also unable to support Ca^2+^‐activated lipid scrambling (Fig. 5B).

We observed that EGFP signals in TMEM16A‐AtSCRD expressing cells were prevalently intracellular (Fig. 5C), indicating that the PM localization of the chimera was impaired to some extent compared to the TMEM16A wild‐type protein. Notably, however, these cells presented clear Alexa555 fluorescence signals along their cell boundaries, when extracellular Ca^2+^ entry was stimulated by ionophore treatment (Fig. 5C). As mentioned above, scrambling activity of mammalian TMEM16E critically depends on serine residue 555 located inside the 35‐aa SCRD [28, 30]. In particular, the Ser555Ile substitution related to limb‐girdle muscular dystrophy [29] was recently shown to cause loss of TMEM16E function [30]. Protein sequence alignments showed that a serine residue is present at the homologous position of AtTMEM16, Ser391 (Fig. 1A). Notably, when the Ser391Ile substitution was introduced into the TMEM16A‐AtSCRD chimera, annexin‐V binding to transfected HEK293 cells was abolished (Fig. 5D). These results suggest that the AtTMEM16 SCRD was sufficient to confer lipid scrambling activity on the TMEM16A chloride channel, which relied on the presence of the conserved Ser391 residue.

### Rescue of a gain‐of function phenotype by a single mutation in the AtTMEM16 SCRD

**Fig. 6:**
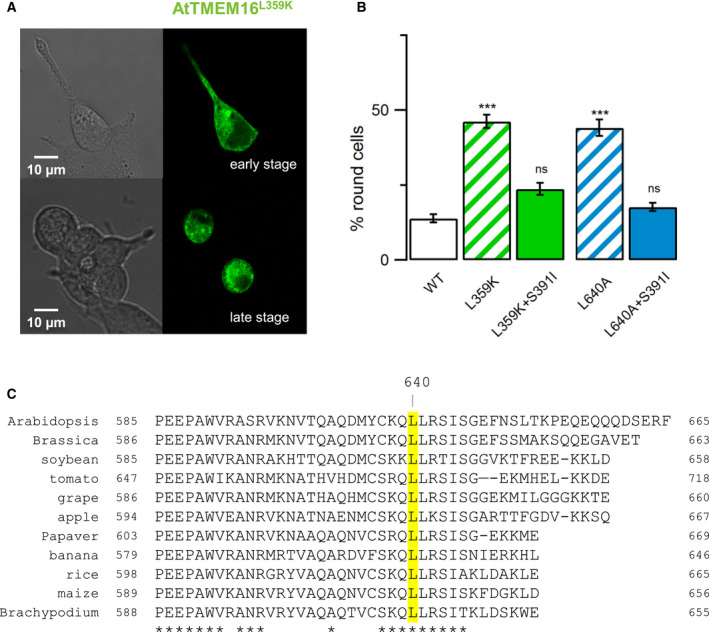
AtTMEM16 gain‐of‐function mutants cause cellular phenotypes reminiscent of aberrant lipid scrambling activity. (A) Confocal images of HEK293 cells transiently transfected with the AtTMEM16^L359K^‐EGFP mutant construct: early‐stage cells (top) were morphologically normal, late‐stage cells (bottom) were round‐shaped and had decreased cell adhesion. (B) Quantification of round‐shaped cells (percentage of EGFP‐positive cells) at 24–30 h after transfection with wild‐type AtTMEM16 (*n* = 17), AtTMEM16^L359K^ (*n* = 19), AtTMEM16^L359K/S391I^ (*n* = 6), AtTMEM16^L640A^ (*n* = 17) or AtTMEM16^L640A/S391I^ (*n* = 14). Data represent mean values ± sem derived from two to five independent transfections. ANOVA/ Tukey's test versus wild‐type AtTMEM16: ***P < 10^−8^ for AtTMEM16^L359K^, *P* = 0.10 for AtTMEM16^L359K/S391I^, ***P < 10^−8^ for AtTMEM16^L640A^, *P* = 0.69 for AtTMEM16^L640A/S391I^. ns, non‐significant. (C) Protein sequence alignment of the C‐terminal regions of TMEM16 homologs from *Arabidopsis thaliana*, *Brassica rapa* (*Brassicaceae*), *Glycine max* (soybean; *Fabaceae*), *Solanum lycopersicum* (tomato; *Solanaceae*), *Vitis vinifera* (grape; *Vitaceae*), *Malus domestica* (apple; *Rosaceae*), *Papaver somniferum* (*Papaveraceae*), *Musa acuminata* (banana; *Musaceae*), *Oryza sativa* (rice), *Zea mays* (maize) and *Brachypodium distachyon* (all from *Poaceae*). Leucine residues homologous to Leu640 in AtTMEM16 are marked in yellow. Highly conserved amino acid residues (> 80% identical) are indicated by an asterisk.

Finally, we used a novel type of functional read‐out for lipid scrambling activity of AtTMEM16, based on the incidence of a round‐shaped phenotype among transfected HEK293 cells [30]. Several earlier studies have shown that cultured HEK293 or COS‐7 cells transiently expressing TMEM16 mutants with constitutive scrambling activity changed their morphology from elongated‐fusiform to round‐shaped and lost their surface adhesion [15, 30, 36, 39]. All tested *TMEM16E* gain‐of‐function mutations causing the human bone disorder gnathodiaphyseal dysplasia (GDD) correlated with high percentages of round‐shaped HEK293 cells, a phenotype which could be rescued by the additional introduction of a loss‐of‐function mutation [30]. Regrettably, none of the amino acid residues affected in TMEM16E GDD mutants is conserved in the AtTMEM16 protein sequence. Therefore, in order to create a hyperactive AtTMEM16 protein, we first made use of the recent identification of an inner activation gate in TMEM16 scramblases and ion channels, constituted by hydrophobic amino acid residues in the middle of the lipid permeation pathway [36]. Interestingly, changing Phe518 in murine TMEM16F (or Leu543 in murine TMEM16A) into a positively charged lysine residue promoted constitutive lipid scrambling activity independent of cytosolic Ca^2+^ binding [36]. AtTMEM16 carries a leucine residue (Leu359) at the homologous position (Fig. 1A). Upon transfection with an AtTMEM16‐EGFP construct carrying the Leu359Lys mutation, we observed that EGFP‐positive HEK293 cells gradually changed from morphologically normal in an early stage (Fig. 6A, top) to a round‐shaped and detached phenotype in later stages (Fig. 6A, bottom). In the time window 24–30 h after transfection, the percentage of round‐shaped cells among all transfected cells was 46 ± 2% on average (Fig. 6B), compared to 14 ± 1% for wild‐type AtTMEM16, which was very close to the value determined for wild‐type TMEM16E [30]. We observed a similar round‐shaped cell percentage for a second mutant, Leu640Ala (44 ± 3%; Fig. 6B). The Leu640 residue is located in the middle of a 9‐aa stretch in the AtTMEM16 C‐terminus, which is highly conserved among plant TMEM16 proteins (Fig. 6C). Elevated round‐cell percentages‐indicative of aberrant scrambling activity ‐ for the Leu640Ala mutant suggested that the C‐terminal region may have an as yet unidentified regulatory role in TMEM16 protein activity. This is also supported by the fact that serine substitution of Phe930 in the C‐terminus of human TMEM16A gave rise to constitutively active chloride channels [40]. Notably, in both Leu359Lys and Leu640Ala mutants, the additional introduction of the Ser391Ile ‘loss‐of‐function’ mutation into the respective mutant background effectively suppressed the round cell phenotype (L359K/S391I: 24 ± 2%; L640A/S391I: 18 ± 1%; Fig. 6B), indicating that modified AtTMEM16 was responsible for aberrant lipid scrambling in intracellular compartments and required the conserved Ser391 residue in its SCRD for this activity.

## Conclusions

The data presented here suggest that AtTMEM16, a plant homolog of eukaryotic TMEM16 proteins, possesses lipid scrambling activity, taking place in intracellular compartments, presumably in the endoplasmic reticulum. Mammalian TMEM16 scramblases with similar localization are TMEM16E [15, 20], TMEM16H [41], and TMEM16K [14], yet our picture of their specific roles in cellular physiology is still blurred. One possibility is that they contribute to the maintenance of membrane symmetry in the ER, where phospholipids are newly synthesized at the cytoplasmic leaflet. In keeping with this idea, the presence of TMEM16K was required for Ca^2+^‐induced phosphatidylserine redistribution within the ER membrane of mouse fibroblasts [42]. Intriguingly, recent studies further suggested tethering roles for TMEM16H at ER/ PM contact sites [41] and TMEM16K at the ER/ endosome interface [43]. Also in plant cells, the ER forms copious connections with other organelles [44], and the subcellular distribution of lipids, membrane signalling at the nano level and interorganellar communication are active fields of research [45, 46]. With the identification of a probable lipid scrambling activity for AtTMEM16, a new player has entered the field. Pollen tube protoplasts from *tmem16* knockout plants showed altered anion currents [26], a phenotype possibly due to indirect effects. In the light of the findings presented here, it will be interesting to screen the knockout plants for specific defects related to protein trafficking, lipid homeostasis and signalling.

## Materials and methods

### Sequence alignments

Protein sequences were aligned using Clustal Omega 1.2.4 (at https://www.ebi.ac.uk/Tools/msa/clustalo/). Human TMEM16 proteins were TMEM16A (986 aa; NCBI Reference Sequence NP_060513.5), TMEM16B (1003 aa; NP_001265525.1), TMEM16E (913 aa; NP_998764.1), TMEM16F (931 aa; NP_001191732.1) and TMEM16K (660 aa; NP_001333397.1). Fungal TMEM16 proteins were afTMEM16 from *Aspergillus fumigatus* (735 aa; XP_746483.1) and nhTMEM16 from *Nectria haematococca* (735 aa; XP_003046028.1). Plant TMEM16 protein sequences were from *Arabidopsis thaliana* (665 aa; NP_001185388.1), *Brassica rapa* (663 aa; XP_009105957.1), *Glycine max* (658 aa; XP_006585539.1), *Solanum lycopersicum* (718 aa; XP_004235618.1), *Vitis vinifera* (666 aa; XP_010654563.1), *Malus domestica* (667 aa; XP_008380823.2), *Papaver somniferum* (669 aa; XP_026449203.1), *Musa acuminata* (659 aa; XP_018679018.1), *Oryza sativa* (665 aa; XP_015621866.1), *Zea mays* (656 aa; NP_001168598.1) and *Brachypodium distachyon* (655 aa; XP_003567040.1). Figures were prepared with jalview 2.11.1.3 software [47].

### cDNA constructs

The full‐length *AtTMEM16* coding sequence was amplified from *Arabidopsis thaliana* Col‐0 cDNA. Total RNA was isolated from leaves of 12‐week‐old Col‐0 plants (SV Total RNA Isolation System, Promega, Milano, Italy) and retro‐transcribed into cDNA. The PCR‐amplification product (oligonucleotides P1‐fw and P1‐rv; Table S1) was subcloned into the pGEM‐T Easy vector (Promega). Sequence analysis confirmed its identity with the At1g73020 gene coding sequence (GenBank NM_105960) deposited at TAIR (The Arabidopsis Information Resource).

For transient expression in Arabidopsis protoplasts, translational fusion constructs with C‐terminal EGFP and N‐terminal YFP were prepared. For the EGFP fusion, the *AtTMEM16* coding sequence was PCR amplified without its stop codon using the oligonucleotides P1‐fw and P2‐rv. This modified *AtTMEM16* ORF was inserted into the unique EcoRI cloning site of the plant expression vector pSAT6‐EGFP‐N1 [48]. For the YFP fusion, the *AtTMEM16*coding sequence was PCR amplified using the oligonucleotides P2‐fw and P1‐rv. The EcoRI‐digested PCR product was inserted into the unique EcoRI cloning site of the plant expression vector pSAT3228‐YFP (kindly provided by Alex Costa, University of Milan, Italy), which puts the *AtTMEM16* ORF in frame with YFP. The correct orientation of the insert was verified by restriction digest and ORF continuity was confirmed by sequencing. For transient expression in HEK293 cells, the *AtTMEM16‐EGFP* cassette in the pSAT6‐derived expression plasmid was PCR amplified using the oligonucleotides P3‐fw and P3‐rv and inserted into NotI/XbaI‐digested pFROG expression vector [49].

Human TMEM16E was expressed using the pFROG‐TMEM16E_898_‐EGFP construct [15], mouse TMEM16A (isoform ac) using pEGFP‐TMEM16A plasmid [50].

The chimeric TMEM16A cDNA carrying the SCRD of AtTMEM16 (amino acids 366‐400) was constructed using an overlap extension PCR approach. The stretch encoding AtTMEM16 SCRD was amplified using the oligonucleotides T16A_atSCRD_fw and T16A_atSCRD_rv (PCR I). The TMEM16A regions upstream and downstream of the SCRD were amplified using the oligonucleotide pairs T16A_5AclI‐fw/atSCRD_T16A‐rv (PCR II) and atSCRD_T16A‐fw/T16A_3BspEI‐rv (PCR III) respectively. The AtTMEM16 SCRD was linked to the TMEM16A upstream region using the products of PCR I and II as template and the oligonucleotide pairs T16A_5AclI_fw/T16A_atSCRD_rv (PCR IV). The TMEM16A downstream region was then added using the products of PCR III and IV as template and the oligonucleotide pairs T16A_5AclI_fw/T16A_3BspEI‐rv. Finally, the resulting chimeric DNA fragment was inserted into the AclI/BspEI restriction sites of the pEGFP‐TMEM16A plasmid, replacing the native TMEM16A fragment.

Single amino acid exchanges were generated using specifically designed oligonucleotides and the QuickChange Lightning site‐directed mutagenesis kit (Agilent Technologies, Cernusco Sul Naviglio, Italy). Mutagenesis was confirmed by DNA sequencing.

### Protoplast isolation and transient transformation

Plants of *Arabidopsis thaliana* (ecotype Columbia‐0) were grown on soil in a growth chamber at 22 °C and under an 8 h light/ 16 h dark regime. Mesophyll protoplasts were isolated from well‐expanded rosette leaves of 4‐ to 8‐week‐old plants. Leaf tissue was digested in enzyme solution containing 1% cellulase and 0.2% macerozyme for 3 h at 23 °C. The protoplast suspension was filtered through a 50‐μm nylon mesh, washed and used for polyethylene glycol (PEG) transformation at a density of 2 × 10^5^ cells ml^−1^ [51, 52]. Protoplasts transformed with either pSAT6‐AtTMEM16‐EGFP or pSAT3228‐YFP‐AtTMEM16 plasmid DNA were then maintained in W5 solution (in mm: 125 CaCl_2_, 154 NaCl, 5 KCl, 2 MES‐KOH pH 5.6) supplemented with 50 μg ml^−1^ ampicillin for up to 3 days at 23 °C in the dark. For co‐localization studies with the ER marker mCherry‐HDEL, protoplasts were co‐transformed with the plasmid construct pCMU‐ERr [53].

### HEK293 cell culture and transfection

Human embryonic kidney (HEK293T) cells were cultured in DMEM supplemented with 10% fetal bovine serum, 100 µg ml^−1^ penicillin, 100 µg ml^−1^ streptomycin and 2 mm l‐glutamine and maintained at 37 °C in a humidified 5% CO_2_/ 95% air atmosphere. Cells were split approximately every 5 days for a maximum of 20 passages. For transient transfection, cells were seeded in a 35‐mm Petri dish and grown until they reached a confluence of about 60%. Transfection was done using 200–400 ng plasmid DNA and Effectene reagent according to the manufacturer's instructions (Qiagen, Milan, Italy). After 8–10 h, cells were split, seeded at low density on glass‐bottom Petri dishes and used for microscopy or patch‐clamp experiments at 24–48 h after transfection.

### Confocal fluorescence microscopy and lipid scrambling assays

Cells were imaged in glass‐bottom Petri dishes (custom‐made or purchased from IBL Baustoff+Labor GmbH, Austria) mounted on a Leica TCS‐SL confocal laser scanning microscope equipped with 40× or 63× oil immersion objectives (numerical aperture 1.25 and 1.45 respectively). Final images are the average of four to eight acquisitions. No filtering was applied. For HEK293 cell imaging, EGFP and FM4‐64 were excited with the 488‐nm laser line and emission acquired at 495–535 nm and 620–800 nm respectively. RFP was excited (sequentially to EGFP) with the 543‐nm laser line and emission acquired at 585–615 nm. For protoplast imaging, EGFP, YFP and FM4‐64 were excited with the 488‐nm laser line and emission acquired at 495–525 nm, 505–535 nm, and 620–800 nm respectively. mCherry was excited with the 543‐nm laser line (sequentially to EGFP/YFP) and emission acquired at 630–680 nm. Red emission signals might contain a contamination by chlorophyll autofluorescence. Chloroplast autofluorescence images were acquired at 700–800 nm.

For a quantitative assessment of AtTMEM16 co‐localization with fluorescently labelled ER marker proteins, we determined Manders' M coefficient representing the fraction of AtTMEM16‐positive pixels containing ER‐marker‐derived signal [54]. M values range from 0 (no overlap) to 1 (complete overlap). Image analysis was done using the JACoP plugin [55] in imagej 1.53 K (at https://imagej.nih.gov/ij/) [56].

In HEK293 cells, endoplasmic reticulum was stained using CellLight ER‐RFP BacMam 2.0 (Thermo Fisher Scientific, Monza, Italy), applied to the Petri dish 36 h before visualization. The PM marker FM4‐64 (Thermo Fisher Scientific) was added to HEK293 cells or protoplasts at a final concentration of 10 μm, and cells were imaged immediately.

For lipid scrambling assays, HEK293 cells were washed in a buffer solution (140 mm NaCl, 2.5 or 5 mm CaCl_2_, 10 mm HEPES, pH 7.4) and incubated with Alexa fluor‐555‐conjugated annexin‐V (Thermo Fisher Scientific), at a dilution of 1 : 100–200, in the absence or presence of the Ca^2+^‐ionophore A23187 (5–10 μm) for 5 min. Ionophore solution was prepared freshly from a 1‐mm stock solution (in DMSO) stored at −20 °C. Alexa fluor‐555 was excited with the 543‐nm laser line and emission acquired at 560–675 nm.

### Patch‐clamp electrophysiology

Current recordings on transiently transfected HEK293 cells were performed in the whole‐cell patch‐clamp configuration, as described elsewhere [15, 30]. The extracellular solution contained (in mm): 140 NaCl, 5 K‐gluconate, 2 CaSO_4_, 2 MgSO_4_, 10 HEPES, pH 7.4. 10 to 30 mm glucose was added to reduce volume‐regulated chloride currents. The intracellular solution with 3 µm free Ca^2+^ contained (in mm): 130 CsCl, 3.209 mm CaCl_2_, 10 HEPES, 10 HEDTA, pH 7.2. Free Ca^2+^ was calculated using the program WinMAXC [57]. Stimulation protocols consisted of voltage steps of 300 ms duration ranging from −80 to +160 mV (with 20‐mV increments), followed by a 175‐ms tail pulse to −80 mV, from a holding potential of 0 mV. Current amplitudes were evaluated at the end of the test pulse, between 280 and 300 ms. Data analysis and figure preparation was done using Ana (freely available at http://users.ge.ibf.cnr.it/pusch/programs‐mik.htm) and Igor pro software (Wavemetrics, Lake Oswego, OR, USA). For the sake of clarity, capacitative transients of current traces were trimmed in the figures.

### Statistical analyses

Data are reported as mean values ± standard error of the mean. Statistical significance was determined using ANOVA followed by a post hoc Tukey’s test to evaluate which data groups showed significant differences. *P* values < 0.05 were considered significant.

## Conflict of interest

The authors declare no conflict of interest.

## Authors’ contributions

AB and JSS conceived the study and designed the experiments. AB, CP, EDZ and JSS performed the experiments. AB, CP and JSS analysed the data. JSS wrote the manuscript.

### Peer review

The peer review history for this article is available at https://publons.com/publon/10.1111/febs.16279.

## Supporting information


**Fig. S1**. Sequence alignment of Arabidopsis and mammalian TMEM16 proteins.
**Table S1**. Oligonucleotides used in this study.

## Data Availability

All data needed to evaluate the conclusions in this work are present in the paper.
