# Supplementary material for: Phospholipid scrambling by a TMEM16 homolog of Arabidopsis thaliana
Source: FEBS J. 2021 Nov 26;289(9):2578–92. doi: 10.1111/febs.16279 (PMC9299152; doi:10.1111/febs.16279)
Supplement: Supplementary file 1 — Fig. S1. Sequence alignment of Arabidopsis and mammalian TMEM16 proteins. Table S1. Oligonucleotides used in this study. [file FEBS-289-2578-s001.zip › febs16279-sup-0001-Supinfo.pdf]

FEBS PRESS
science publishing by scientists

WILEY

The FEBS Journal

www.febsjournal.org

Phospholipid scrambling by a TMEM16 homolog of *Arabidopsis thaliana*

Anna Boccaccio, Cristiana Picco, Eleonora Di Zanni and Joachim Scholz-Starke

DOI: 10.1111/febs.16279

Supplementary Information for

Phospholipid scrambling by a TMEM16 homolog of Arabidopsis thaliana

Anna Boccaccio, Cristiana Picco, Eleonora Di Zanni and Joachim Scholz-Starke*

Institute of Biophysics, Consiglio Nazionale delle Ricerche, Genova, Italy

* To whom correspondence should be addressed:

Joachim Scholz-Starke
E-mail: joachim.scholzstarke@ibf.cnr.it

This PDF file includes:

Figure S1
Table S1
SI References

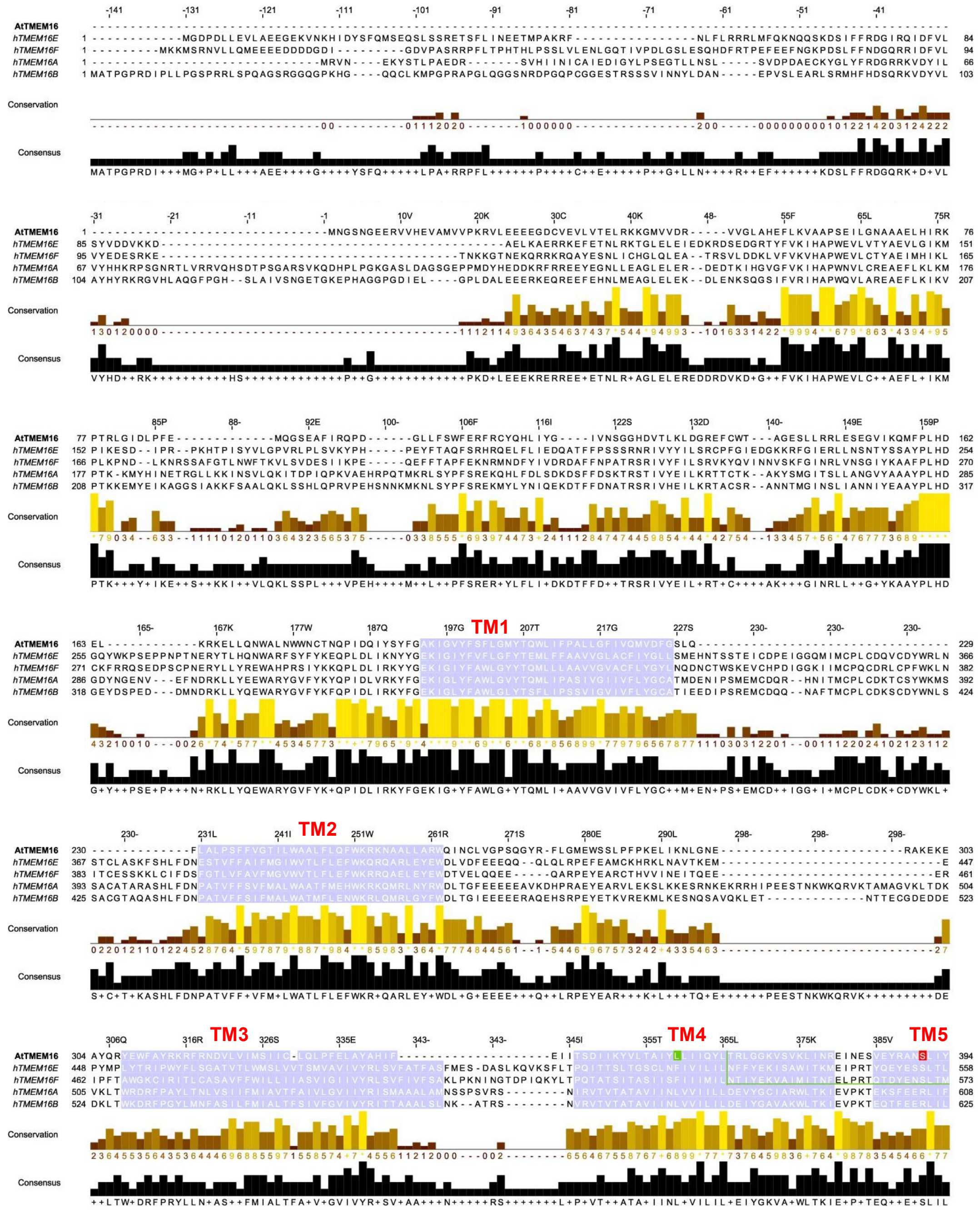

AtTMEM16
hTMEM16E
hTMEM16F
hTMEM16A
hTMEM16B
Conservation
Consensus
TM1
TM2
TM3
TM4
TM5

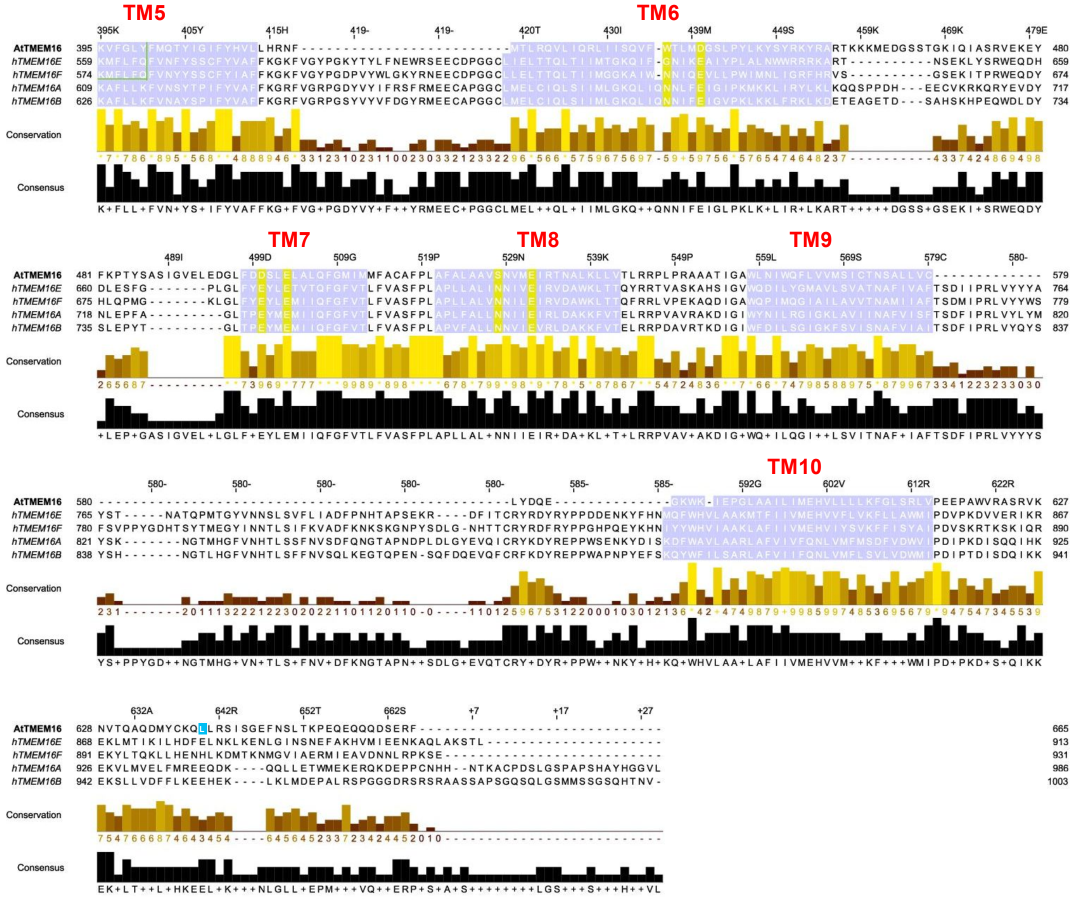

Figure S1: Sequence alignment of Arabidopsis and mammalian TMEM16 proteins.

Protein sequences of AtTMEM16, the human lipid scramblases TMEM16E and TMEM16F and the human Cl^- channels TMEM16A and TMEM16B were aligned using Clustal Omega. Numbering in the top line corresponds to AtTMEM16. Trans-membrane domains based on the murine TMEM16F protein structure [1] are marked in light blue. The SCRD homology region is indicated by a green line. Residues Leu359, Ser391 and Leu640 (mutated in this study) are highlighted in green, red and blue, respectively. The primary Ca^{2+} binding site residues in TM 6-8 [2] are marked in yellow. The figure was prepared using Jalview software. The conservation annotation is a quantitative numerical index reflecting the conservation of the physico-chemical properties for each column of the alignment, with identities scoring highest, and amino acids with substitutions in the same physico-chemical class have next highest score. The score for each column is shown below the histogram. The conserved columns with a score of 11 are indicated by '*'. Columns with a score of 10 (showing amino acid substitutions but all properties are conserved) are marked with a '+'. The consensus annotation reflects the percentage of a given residue per column.

Table S1: Oligonucleotides used this study

Oligo name	5'-3' sequence
P1-fw	CTA GGA ATT CAT GAA TGG GAG TAA TGG GGA GG
P1-rv	CTA GGA ATT CTC AAA AGC GCT CTG AGT CTT GTT G
P2-fw	CTA GGA ATT CTA TGA ATG GGA GTA ATG GGG AGG
P2-rv	CTA GGA ATT CAA AGC GCT CTG AGT CTT GTT GTT G
P3-fw	CTA GGC GGC CGC ATG AAT GGG AGT AAT GGG GAG G
P3-rv	CTA GTC TAG ATC ACT TGT ACA GCT CGT CCA TG
T16A_atSCRD_fw	CTC GTG GTC ATC ATT CTG CTG ACT CGG CTA GGA GGG AAA G
T16A_atSCRD_rv	GAT GGG AGT GTA AGA GTT CAC AAA ATA GAG CCC AAA AAC TTT GTA AAT C
T16A_5AclI-fw	GGG TGA CAA CGT TGA GTT CAA C
T16A_3BspEI-rv	CTC CAT CCG GAA AGA GCG G
atSCRD_T16A-fw	GAT TTA CAA AGT TTT TGG GCT CTA TTT TGT GAA CTC TTA CAC TCC CAT C
atSCRD_T16A-rv	CTT TCC CTC CTA GCC GAG TCA GCA GAA TGA TGA CCA CGA G
TMEM_L359K_fw	AAG TAC GTA TTG ACA GCT ATC TAC **AAG** CTA ATC ATT CAG TAC CTC ACT CGG
TMEM_L359K_rv	CCG AGT GAG GTA CTG AAT GAT TAG **CTT** GTA GAT AGC TGT CAA TAC GTA CTT
TMEM_S391I_fw	GTG TGG AAT ACC GAG CTA ACA **T**CT TGA TTT ACA AAG TTT TTG GG
TMEM_S391I_rv	CCC AAA AAC TTT GTA AAT CAA G**A**T GTT AGC TCG GTA TTC CAC AC
TMEM_L640A_fw	CAC AAG ACA TGT ACT GTA AAC AG**G C**CT TAA GAA GCA TTT CTG GTG AAT
TMEM_L640A_rv	ATT CAC CAG AAA TGC TTC TTA AG**G C**CT GTT TAC AGT ACA TGT CTT GTG

Restriction site sequences are underlined. Nucleotides corresponding to amino acid exchanges are indicated in bold letters.

SI References

1 Alvadia C, Lim NK, Clerico Mosina V, Oostergetel GT, Dutzler R & Paulino C (2019) Cryo-EM structures and functional characterization of the murine lipid scramblase TMEM16F. *eLife* **8**.

2 Brunner JD, Lim NK, Schenck S, Duerst A & Dutzler R (2014) X-ray structure of a calcium-activated TMEM16 lipid scramblase. *Nature* **516**, 207–212.
